# The Role of Iron in *Staphylococcus aureus* Infection and Human Disease: A Metal Tug of War at the Host—Microbe Interface

**DOI:** 10.3389/fcell.2022.857237

**Published:** 2022-03-24

**Authors:** Madeleine C. van Dijk, Robin M. de Kruijff, Peter-Leon Hagedoorn

**Affiliations:** ^1^ Department of Biotechnology, Delft University of Technology, Delft, Netherlands; ^2^ Department of Radiation Science and Technology, Delft University of Technology, Delft, Netherlands

**Keywords:** *Staphylococcus aureus*, MRSA, iron homeostasis, nutritional immunity, iron deficiency anemia, ferric uptake regulator, iron-regulated surface determinant system, heme

## Abstract

Iron deficiency anemia can be treated with oral or intravenous Fe supplementation. Such supplementation has considerable effects on the human microbiome, and on opportunistic pathogenic micro-organisms. Molecular understanding of the control and regulation of Fe availability at the host-microbe interface is crucial to interpreting the side effects of Fe supplementation. Here, we provide a concise overview of the regulation of Fe by the opportunistic pathogen *Staphylococcus aureus*. Ferric uptake regulator (Fur) plays a central role in controlling Fe uptake, utilization and storage in order to maintain a required value. The micro-organism has a strong preference for heme iron as an Fe source, which is enabled by the Iron-regulated surface determinant (Isd) system. The strategies it employs to overcome Fe restriction imposed by the host include: hijacking host proteins, replacing metal cofactors, and replacing functions by non-metal dependent enzymes. We propose that integrated omics approaches, which include metalloproteomics, are necessary to provide a comprehensive understanding of the metal tug of war at the host-microbe interface down to the molecular level.

## Introduction

Iron deficiency is a major health concern worldwide, resulting in over one billion cases of iron-deficiency anemia (Gardner and Kassebaum 2020; Pasricha et al., 2021). Currently, the first-line treatment for iron deficiency anemia is the use of oral iron supplements. However, many side effects have been reported following their use: e.g., 30–70% of the patients report gastrointestinal problems (DeLoughery 2019). The supplemented iron is only partially absorbed by the human body, resulting in a significantly increased amount of iron available to the human gut microbiome (DeLoughery 2019; Finlayson-Trick et al., 2020). The microbiome of an individual plays an important role in human health, and metal compounds are known to affect the survival and reproduction of bacteria (Sheldon and Skaar 2019). Recognition of these side effects and the discovery that intravenously administered iron causes only minor adverse effects, after having been (incorrectly) considered more dangerous for decades, has sparked the use of intravenous Fe supplementation (Auerbach, Gafter-Gvili, and Macdougall 2020; Schaefer et al., 2020). However, following intravenous iron administration, blood borne pathogens will be exposed to excess iron. One of these pathogens, responsible for a wide variety of clinical diseases, is *Staphylococcus aureus* of which the methicillin resistant (MRSA) strain represents a global human health challenge (David and Daum 2010; DeLeo et al., 2010; Ganz et al., 2020).

The effect of supplemented iron on *S. aureus* proliferation has been investigated in a few patient studies only, where bacterial growth assays were performed on serum samples taken from the subjects following iron supplementation. In this way, Cross et al. (2015) found supplemented oral iron to significantly increase transferrin saturation (TSAT) in the serum samples. However, while gram-negative bacteria, including *E. coli*, and the gram-positive *Staphylococcus epidermis,* showed elevated growth rates, *S. aureus* appeared unaffected (Cross et al., 2015). The authors suggested this to be caused by a preference of *S. aureus* for heme iron over transferrin-bound iron (Cross et al., 2015), which is consistent with other studies (Barton Pai et al., 2006; Suffredini et al., 2017; Skaar et al., 2004). In fact, in hemodialysis patients, which have significantly lower transferrin levels, intravenous iron sucrose administration was found to correlate with increased non-transferrin-bound iron (NTBI) levels in the patients’ serum. Significantly increased *S. aureus* growth was observed on the serum samples of these patients, compared to the NTBI-negative subjects (Barton Pai et al., 2006). This indicates that the molecular form of iron in the blood influences its uptake by *S. aureus*, which seems to prefer NTBI and heme iron, but is less responsive to transferrin-bound iron.

While multiple reviews have recently been published on the interaction of supplemented iron and enteric pathogens on a molecular level (Yilmaz and Li 2018; Finlayson-Trick et al., 2020; Qi et al., 2020), investigations focusing on the impact of supplemented intravenous iron on blood borne pathogens such as *S. aureus* are lacking. Therefore, in this mini-review, we aim to give an overview of recent insights into iron and *S. aureus* in the context of excess iron and iron-limiting conditions imposed by the host (nutritional immunity) during *S. aureus* infections. Here, we will first look in detail at the regulation systems *S. aureus* uses to control uptake of both free and heme iron, and to regulate the intracellular Fe levels, and then describe how the pathogen is able to survive under iron starvation conditions.

## Regulation of Iron Homeostasis in *S. aureus* at the Microbe-Host Interface

### Control Systems

In engineering, control systems regulate the operation of devices and their processes using control loops. For a functioning control loop, you need to measure a process value which can be either below or above a target set point. The device or process then needs to be adjusted to attain the desired process value. In a similar manner, bacteria have evolved to control the intracellular concentrations of nutrients and metabolites, including iron, to pre-set conditions required for growth and/or maintenance. For this process, called homeostasis, bacteria produce sensors that measure the amount of intracellular iron, and a control system (or systems) that can change the expression of proteins and the functionality of enzymes in order to reach the target value. This control occurs at: transcriptional (DNA→ mRNA), post-transcriptional (stabilizing or degradation of mRNA), translational (mRNA→ protein) or post-translational level (protein degradation, modification, and allosteric interaction). These four levels allow very precise tuning and distribution of iron, depending on necessity and environmental conditions. Precise tuning is important because Fe is essential for life, while at the same time Fe^2+^ can generate toxic reactive oxygen species (ROS) with O_2_, and Fe^3+^ is insoluble under neutral aqueous conditions.

### Regulation of Free Iron

**FIGURE 1:**
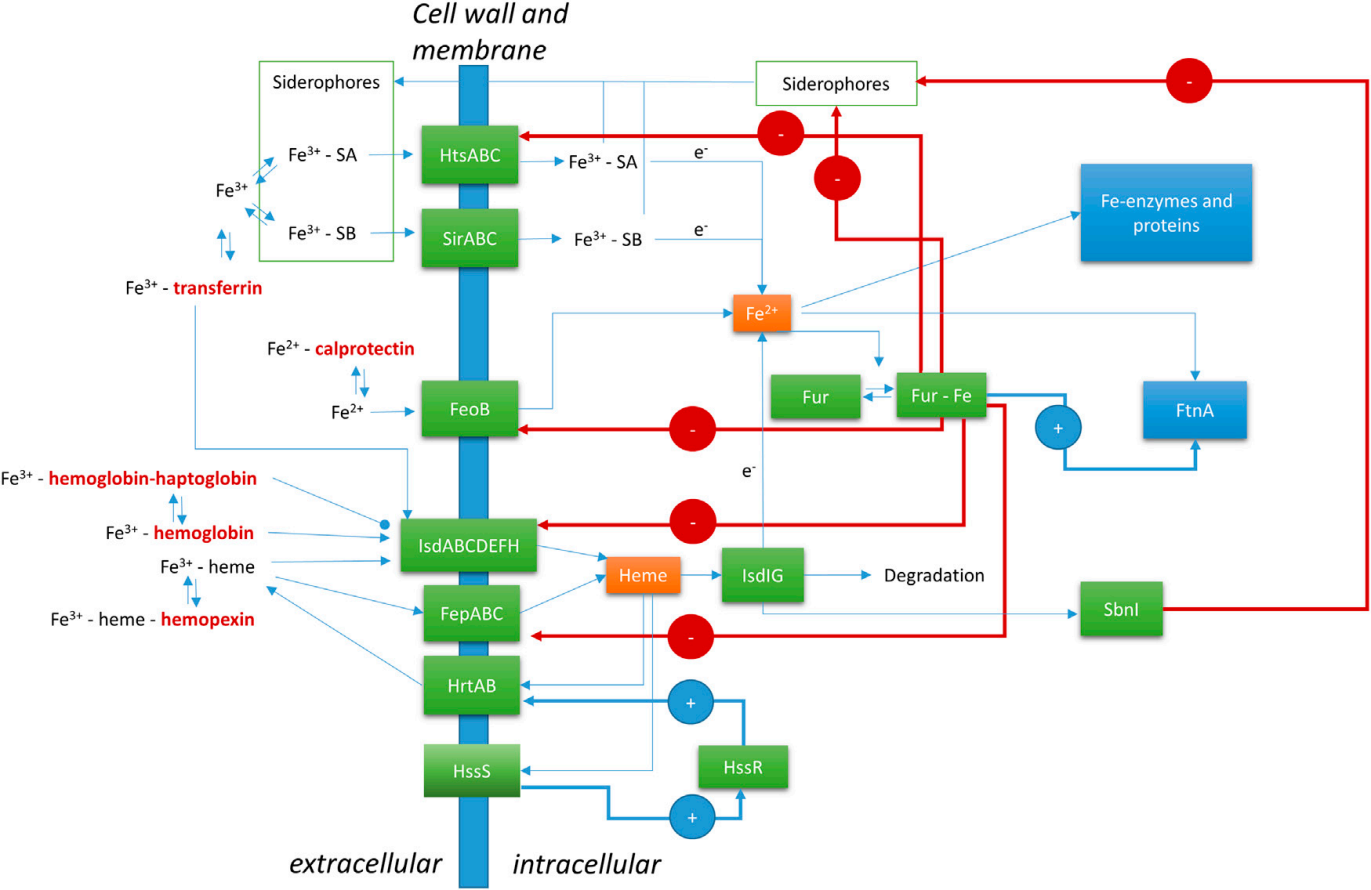
Regulation of Fe by *Staphylococcus aureus* at the host-microbe interface. FeoB, ferrous Fe transporter; FepABC, Fe dependent peroxidase transporter; FtnA, ferritin; Fur, ferric uptake regulator; HrtAB, heme regulator transporter efflux pump; HssR, heme sensing two-component regulator regulatory protein; HssS, heme sensing two-component regulator sensor protein; HtsABC, heme transport system involved in Fe-SA uptake; IsdABCDEFGHI, iron-regulated surface determinant system; SA, Staphyloferrin A; SB, Staphyloferrin B; SbnI, L-serine kinase and heme responsive regulator of SB biosynthesis. Human (host) proteins are in bold and dark red font.

In *Staphylococcus aureus,* Fur (ferric uptake regulator) is the major control system for iron (Figure 1). The Fur protein is homodimeric, with each monomer consisting of an N-terminal DNA binding domain and a C-terminal dimerization domain (Price and Boyd 2020). Between the two domains is a metal ion binding site, which was recently shown to bind a [2Fe-2S] cluster in *E. coli* (Fontenot et al., 2020). *S. aureus* Fur has been described as binding two separate Fe^2+^ ions in the hinge regions between the N- and C-terminal domains. *E. coli* and *S. aureus* Fur share 30% sequence identity and 49% sequence similarity, which includes three conserved Cysteines (Supplementary Material). The precise nature of the Fe-bound form of *S. aureus* Fur remains to be established. Upon dimerization, the DNA binding part of Fe-bound Fur is a transcriptional repressor of a range of genes related to iron homeostasis. It functions by binding to a so-called Fur-box upstream of the coding genes. These genes involve Fe transporters and many other genes, as discussed below. Some are established virulence factors, which means they are involved in disease processes. Related proteins, called Fur family proteins, with affinity to other metal ions or compounds have been discovered, such as Zur (zinc uptake regulator) for Zn^2+^ and PerR (peroxide operon regulator) which is a metal-dependent regulator for hydrogen peroxide. The Fe^2+^ and Mn^2+^ dependence of PerR highlights crosslinks between the different control systems (Horsburgh, Ingham, and Foster 2001).

Ferrous iron uptake by transporter FeoB is still poorly characterized, although recently inhibitors have been identified that may have important medical implications as novel antibiotics against MRSA and other multidrug resistant Gram-positive bacteria (Shin et al., 2021). Fur and PerR differentially regulate the *S. aureus* ferritin gene (FtnA), which encodes the Fe-storage protein ferritin (Morrissey et al., 2004). Ferritin can take up circa 4,000 Fe atoms in the form of a ferrihydrite mineral nanoparticle inside a 24-meric protein sphere (Honarmand Ebrahimi, Hagedoorn, and Hagen 2015). Iron storage by ferritin involves oxidation of Fe^2+^ to Fe^3+^ and concerted incorporation into a growing ferrihydrite mineral core. Upon reduction, by a mechanism that has not yet been established, Fe can also be released from ferritin as Fe^2+^.

Another Fur controlled Fe uptake system is the FepABC (Fe dependent peroxidase) transporter. The transporter has not been well characterized, but it has been implicated in Fe and possibly heme uptake. The transporter consists of FepA, a predicted membrane anchored lipoprotein that may act as an Fe (compound) binding protein, peroxidase FepB, and integral membrane protein FepC (Biswas et al., 2009). FepB can bind heme and protoporphyrin IX (heme without Fe) and has a low peroxidase activity (Turlin et al., 2013). FepB is a substrate of the Twin-Arginine Translocation pathway, which allows membrane translocation of fully folded cofactor bound proteins (Biswas et al., 2009). Heterologous expression of the *S. aureus* FepAB in *E. coli* allowed heme utilization in this organism (Turlin et al., 2013).

Siderophores are small, extracellular, peptide-derived compounds with a high affinity for Fe^3+^. *S. aureus* produces two siderophores: Staphyloferrin A (SA) and Staphyloferrin B (SB). SA is produced using the gene cluster *sfa*, and SB is produced using the *sbn* gene cluster (Marchetti et al., 2020). The gene clusters are both transcriptionally repressed by Fur. Recently, a heme sensitive regulator of siderophore production was identified: SbnI (Laakso et al., 2016; Verstraete et al., 2019). The gene product of SbnI is an enzyme producing a precursor to the siderophore. Furthermore, the protein can bind DNA and upon dimerization contains a heme binding domain. Heme transfer from IsdI to SnbI has been suggested to help control the production of siderophores, thereby shifting focus to heme utilization rather than free iron uptake. Fe^3+^ bound SA is taken up using the ABC transporter HtsABC (heme transport system). Despite the name, it is unclear if the Hts transporter is involved in heme uptake, and if so by which mechanism (Price and Boyd 2020). Hts transcription is regulated by Fur. SB is taken up by the ABC transporter SirABC (Grigg et al., 2010a).

Siderophores play a very important role in *Staphylococcus* biofilm formation to ensure Fe availability (Johnson et al., 2005; Oliveira et al., 2021). Fe chelators that compete with siderophores can disturb biofilm formation and may therefore be of medical importance (Richter et al., 2017; Coraça-Huber et al., 2018).

### Regulation of Heme Iron

Heme obtained from red blood cells is a major source of iron for *Staphylococcus aureus* during infections (Skaar et al., 2004). There is evidence that *S. aureus* has evolved a specificity towards human hemoglobin versus other mammalian orthologs that is unique among pathogenic bacteria (Pishchany et al., 2010). However, a high level of intracellular heme is dangerous due to its potential to form reactive oxygen species (ROS). *S. aureus* uses the two-component regulator HssRS (Figure 1) (Heme sensing two-component system) to control the intracellular level of free heme (Price and Boyd 2020; Stauff, Torres, and Skaar 2007; Stauff and Skaar 2009). HssS is a transmembrane protein, which responds to the heme level by an unknown mechanism. Upon activation, HssS acts as a histine kinase to phosphorylate the histidine of HssR, thereby activating the protein as a transcriptional activator of the heme efflux transporter HrtAB (Heme regulator transporter efflux pump). Whether the precise compound that is expelled by HrtAB is heme or a heme metabolite is unknown (Price and Boyd 2020). Additional targets of HssR have not been identified to date.

The cell wall of *S. aureus* contains a unique system to acquire heme, which is called Isd (Iron-regulated surface determinant system) (Skaar and Schneewind 2004; Grigg et al., 2010b; Mazmanian et al., 2003). Similar systems are present in other Gram-positive pathogenic bacteria, such as *Listeria monocystogenes* and *Clostridium tetani*. This system takes up heme from human (host) hemoproteins. The Isd system involves nine different proteins, of which four are bound to the cell wall: IsdA, IsdB, IsdC, and IsdH. Two proteins, IsdE and IsdF, constitute an ABC-transporter for the heme cofactor. IsdD is a transmembrane protein of unknown function. And the final two proteins, IsdI and IsdG, are soluble intracellular heme degrading enzymes. The outer cell wall proteins IsdB and IsdH bind free heme, methemoglobin and hemoglobin-haptoglobin complexes from the host. The cell wall proteins IsdC and IsdA are involved in heme transport through the 15–30 nm thick cell wall to the ligand binding component of the ABC-transporter IsdE. After translocation of the heme to the cytoplasm, the cofactor is degraded by the heme degrading enzymes IsdI and IsdG. These enzymes are distantly related to well-characterized heme oxygenases and have been found to release Fe from the cofactor, yet the precise reaction mechanism remains to be solved (Grigg et al., 2010b). Fur regulates the expression of the genes for IsdA, IsdB, IsdC and IsdH. The gene for the enzyme sortase B (SrtB) is part of the same transcriptional unit as IsdC, and therefore also regulated by Fur. Sortase B is involved in the cell wall anchoring of the Isd components.

## Overcoming Reduced Iron Availability

### Strategies

Upon infection, the host starts the immune response. Macrophages are activated by interaction with *S. aureus via* Toll-like receptors (TLRs) (Pidwill et al., 2021). This starts signal transduction cascades which include mechanisms to limit the availability of iron in blood (Pandur et al., 2021). Central in the regulation of these processes is the hormone hepcidin. Hepcidin interacts with the host Fe efflux protein ferroportin, thereby limiting Fe export from macrophages (Theurl et al., 2008). Interestingly the same exposed *S. aureus* lipoproteins that support Fe acquisition, for example, *via* the Isd system, are also recognized by the TLRs, thereby evoking inflammation responses in the host (Schmaler et al., 2009; Sheldon and Heinrichs 2012). One of the cellular host responses involved is the endocytosis and degradation of erythrocytes by the macrophages in a process called erythrophagocytosis (Knutson et al., 2005). The Fe retained by the host cells is also put to good use, as Fe^2+^ enabled production of ROS is used to kill bacteria taken up by these cells (Rosen et al., 1995; Haschka, Hoffmann, and Weiss 2021). Interestingly. ROS also induce antibiotic resistance in *S. aureus*, indicating a negative side-effect of our innate immune response (Rowe et al., 2020).

In the context of sepsis, *S. aureus* is capable of lysing erythrocytes by secreting hemolytic toxins to free hemoglobin and obtain it through the Isd system (Torres et al., 2010). The *S. aureus* heme-oxygenases IsdG and IsdI have been shown to be important for full virulence with heme as the primary iron source (Reniere and Skaar 2008). Host heme oxygenase 1 (HO1) catalyzes the rate-limiting step in heme degradation, producing biliverdin, Fe^2+^ and CO (carbon monoxide) (Singh et al., 2018). CO can act as a messenger in various protective cascades. Links between HO1 and protective effects on *S. aureus* infection have been shown (MacGarvey et al., 2012; Gahlot et al., 2017).

The host restricts the availability of iron in its different forms further by producing the hemoglobin binding protein haptoglobin, the heme binding protein hemopexin, the free Fe^3+^ binding proteins transferrin and lactoferrin, and the free Fe^2+^ binding protein calprotectin (Nakashige et al., 2015; Marchetti et al., 2020). Haptoglobin binding to hemoglobin inhibits uptake of heme by the Isd system of *S. aureus*, although the protein still binds to IsdH (Mikkelsen, Runager, and Andersen 2020). Calprotectin (CP) was originally identified to be involved in Mn^2+^ limitation by the host, but was more recently found to bind Fe^2+^ efficiently in the presence of Ca^2+^ (Nakashige et al., 2015). It has been demonstrated that CP induces Fe starvation in *S. aureus* cultures (Obisesan, Zygiel, and Nolan 2021; Zygiel et al., 2021). The ability of *S. aureus* to efficiently incorporate heme affords protection against CP induced Fe starvation (Zygiel et al., 2021). In the preceding sections we have described the mechanisms through which *S. aureus* controls intracellular Fe levels in response to iron sources in the human host. However, these control systems may be insufficient when Fe availability is strongly reduced. Pathogenic bacteria such as *S. aureus* have evolved several strategies to tackle the metal restrictions imposed through nutritional immunity: 1) hijacking host proteins, 2) replacing metal cofactors, and 3) replacing functions by non-metal dependent enzymes. We will discuss examples of each strategy below.

### Hijacking Host Proteins

*S. aureus* cannot use hemopexin as a heme source using the Isd system. However, it has been reported to take up iron from host transferrin using a transferrin receptor. The nature of the transferrin receptor of *S. aureus* is convoluted in literature. This cell-wall associated protein was first identified as a functional glyceraldehyde-3-phosphate dehydrogenase (GAPDH) (Modun, Morrissey, and Williams 2000). However, this was shown to be incorrect, and the protein was identified as staphylococcal transferrin-binding protein StbA (Taylor and Heinrichs 2002). Later, it was shown that StbA is the same protein as IsdA, part of the Isd system for heme uptake described above (Clarke, Wiltshire, and Foster 2004; Maresso and Schneewind 2006). The ongoing tug of war for Fe between host transferrin and bacterial transferrin receptors has caused rapid evolutionary development of the involved proteins (Barber and Elde 2014). The presence of a transferrin receptor indicates that *S. aureus* can take up Fe from transferrin, at least to some extent, although the major Fe source from the host is heme.

### Replacing Metal Cofactors

A well-established example of replacing metal cofactors by *S. aureus* are the Mn-dependent superoxide dismutases (SODs). Neutrophils, and other host immune cells, can induce oxidative bursts as a defensive strategy against *S. aureus* (Rigby and DeLeo 2012). This process generates high levels of damaging ROS, including superoxide (Forrester et al., 2018; Jakubczyk et al., 2020). The expression of SODs is one way in which *S. aureus* can combat ROS. *S. aureus* has two superoxide dismutases, SodA and SodM. SodA can incorporate only Mn as metal cofactor while SodM can use either Fe or Mn depending on the conditions (Garcia et al., 2017). As part of the host immune response, the neutrophil protein CP sequesters trace metals, including Mn. The action of CP disturbs the correct metalation of SodA (Kehl-Fie et al., 2013). However, *S. aureus* encodes an additional Mn-dependent SOD, SodM, which can substitute its metal cofactor for Fe under Mn-limiting conditions (Garcia et al., 2017; Treffon et al., 2020). In this way, *S. aureus* can retain sufficient SOD activity despite CP activity and maintain virulence. Small non-coding regulatory RNA molecule RsaC (co-transcribed with Mn transporter MntABC) represses the translation of the SodA coding mRNA under Mn limiting conditions. So, if there is a shortage of Mn (Mn uptake by MntABC needed), the Mn SOD is suppressed in favor of the Fe containing SOD. A clinically relevant example of a highly oxidative stressful environment with strong CP presence are the airways of cystic fibrosis patients, where *S. aureus* can cause persistent infections for years. Investigation of gene expression demonstrated significantly elevated SodM expression levels in clinical isolates compared to laboratory strains (Treffon et al., 2020).

### Replacing Functions by Non-metal Dependent Enzymes

An alternative strategy of *S. aureus* to respond to nutritional immunity is to use a protein variant that lacks a metal cofactor altogether. The consumption of glucose through glycolysis is a process fundamental to many life forms. For bacteria, some of the enzymes involved are Mn-dependent. Yet even under Mn-limited conditions, *S. aureus* was shown to prefer glucose as main carbon source despite its burden on cellular Mn demand (Radin et al., 2019b). It has recently been demonstrated that *S. aureus* can express a Mn-independent variant of phosphoglycerate mutase to maintain glucose consumption under Mn-stress (Radin et al., 2019a). The discovery of metal-independent variants is not unique to *S. aureus* and may indicate a broader pattern among bacteria.

## Conclusion and Perspectives

Despite the wealth of knowledge on metal homeostasis in *S. aureus* at the host-microbe interface, a comprehensive overview of the complex interactions between different metal control systems is currently lacking. Many individual proteins and pathways have been identified. However, in most cases, the molecular mechanisms of action of all these different proteins are not fully established. We envision that an integrated omics approach to determine the changes in the proteome, metabolome and metalloproteome will offer a comprehensive view of the pathogen response to nutritional immunity as well as Fe overload. Recently, integrated omics approaches have been successfully used to explore the molecular action of metallodrugs such as platinum and ruthenium anticancer drugs (Wang, Li, and Sun 2019; Steel and Hartinger 2020). Of key importance will be metalloproteomics, which will provide information on the changing intracellular metal distribution among *S. aureus*’ proteins. Such experiments have been performed for Fe in *E. coli* using the metalloproteomic approach Metal Isotope Radio Autography in Gel Electrophoresis (MIRAGE), which showed substantial changes in Fe distribution among proteins under normal and high Fe conditions (Sevcenco et al., 2011; 2009). It will be especially interesting to focus on the complex interplay between Fe and Mn homeostasis in this respect, as described above for SOD. A comprehensive understanding down to the molecular level will provide a new basis for the development of treatments against pathogenic bacteria, including MRSA. At the same time, it will allow clinicians to take pathogen response into account when treating iron-deficient patients, paving the way towards personalized treatment. This new understanding is crucial, especially considering the continuous increase of multidrug-resistant bacteria and their far-reaching impact on human health globally.
