# Supplementary material for: The Role of Iron in Staphylococcus aureus Infection and Human Disease: A Metal Tug of War at the Host—Microbe Interface
Source: Front Cell Dev Biol. 2022 Mar 24;10:857237. doi: 10.3389/fcell.2022.857237 (PMC8986978; doi:10.3389/fcell.2022.857237)
Supplement: Supplementary file 1 [file DataSheet1.pdf]

Supplementary Material

Supplementary Data

AA sequences. Cysteines indicated in red and bold font.

E. coli Fur (UniProt ID P0A9A9)

MTDNNTALKKAGLKVTLPRLKILEVLQEPDNHHVSAEDLYKRLIDMGEEIGLATVYRVLNQFDDAGI
VTRHNFEGGKSVFELTQQHHHDHLI**C**LD**C**GKVIEFSDDSIEARQREIAAKHGIRLTNHSLYLYGH**C**A
EGD**C**REDEHAHEGK

S. aureus Fur (UniProt ID 5HFK6)

MNTNDAIKILKENGLKYTDKRKDMLDIFVEEDKYINAKYIQQVMDENYPGISFDTIYRNLHLFKDLG
IIENTELDGEMKFRIA**C**TNHHHHHFI**C**EKCGDTKVIDY**C**PIDQIKLSLPGVNIHKHKLEVYGV**C**ES**C**
QD

Sequence alignment (BlastP, RRID:SCR001010); 30% identity, 49% similarity, 6% gaps

```
SaFur  10   LKENGLKYTDKRKDMLDIFVEEDKY-INAKYIQQVMDENYPGISFDTIYRNLHLFKDLGI  68
            LK+ GLK T  R  +L++  E D + ++A+ + + + +    I   T+YR L+ F D GI
EcFur  8    LKKAGLKVTLPRLKILEVLQEPDNHHVSAEDLYKRLIDMGEEIGLATVYRVLNQFDDAGI  67

SaFur  69   IENTELDGEMKFRIACTNHHHHHFICEKCGDTKVIDYC--PIDQIKLSLP---GVNIHKH  123
            +     +G          HHH H IC  CG  KVI++    I+  +  +    G+ +  H
EcFur  68   VTRHNFEGGKSVFELTQQHHHDHLICLDCG--KVIEFSDDSIEARQREIAAKHGIRLTNH  125

SaFur  124  KLEVYGVC  131
             L +YG C
EcFur  126  SLYLYGHC  133
```
